# Prevalence of Perceived Stress, Anxiety, Depression, and Obsessive-Compulsive Symptoms in Health Care Workers and Other Workers in Alberta During the COVID-19 Pandemic: Cross-Sectional Survey

**DOI:** 10.2196/22408

**Published:** 2020-09-25

**Authors:** Kelly Mrklas, Reham Shalaby, Marianne Hrabok, April Gusnowski, Wesley Vuong, Shireen Surood, Liana Urichuk, Daniel Li, Xin-Min Li, Andrew James Greenshaw, Vincent Israel Opoku Agyapong

**Affiliations:** 1 Strategic Clinical Networks™ Provincial Clinical Excellence Alberta Health Services Calgary, AB Canada; 2 Department of Psychiatry Faculty of Medicine and Dentistry University of Alberta Edmonton, AB Canada; 3 Cumming School of Medicine University of Calgary Calgary, AB Canada; 4 Addiction and Mental Health Alberta Health Services Edmonton, AB Canada

**Keywords:** health care worker, COVID-19, pandemic, mental health, depression, anxiety, stress, obsessive compulsive

## Abstract

**Background:**

During pandemics, effective containment and mitigation measures may also negatively influence psychological stability. As knowledge about COVID-19 rapidly evolves, global implementation of containment and mitigation measures has varied greatly, with impacts to mental wellness. Assessing the impact of COVID-19 on the mental health needs of health care workers and other workers may help mitigate mental health impacts and secure sustained delivery of health care and other essential goods and services.

**Objective:**

This study assessed the self-reported prevalence of stress, anxiety, depression, and obsessive-compulsive symptoms in health care workers and other workers seeking support through Text4Hope, an evidence-based SMS text messaging service supporting the mental health of residents of Alberta, Canada, during the COVID-19 pandemic.

**Methods:**

An online cross-sectional survey gathered demographic (age, gender, ethnicity, education, relationship, housing and employment status, employment type, and isolation status) and clinical characteristics using validated tools (self-reported stress, anxiety, depression, and contamination/hand hygiene obsessive-compulsive symptoms). Descriptive statistics and chi-square analysis were used to compare the clinical characteristics of health care workers and other workers. Post hoc analysis was conducted on variables with >3 response categories using adjusted residuals. Logistic regression determined associations between worker type and likelihood of self-reported symptoms of moderate or high stress, generalized anxiety disorder, and major depressive disorder, while controlling for other variables.

**Results:**

Overall, 8267 surveys were submitted by 44,992 Text4Hope subscribers (19.39%). Of these, 5990 respondents were employed (72.5%), 958 (11.6%) were unemployed, 454 (5.5%) were students, 559 (6.8%) were retired, 234 (2.8%) selected “other,” and 72 (0.9%) did not indicate their employment status. Most employed survey respondents were female (n=4621, 86.2%). In the general sample, the 6-week prevalence rates for moderate or high stress, anxiety, and depression symptoms were 85.6%, 47.0%, and 44.0%, respectively. Self-reported symptoms of moderate or high stress, anxiety, and depression were all statistically significantly higher in other workers than in health care workers (*P*<.001). Other workers reported higher obsessive-compulsive symptoms (worry about contamination and compulsive handwashing behavior) after the onset of the pandemic (*P*<.001), while health care worker symptoms were statistically significantly higher before and during the COVID-19 pandemic (*P*<.001). This finding should be interpreted with caution, as it is unclear the extent to which the adaptive behavior of health care workers or the other workers might be misclassified by validated tools during a pandemic.

**Conclusions:**

Assessing symptoms of prevalent stress, anxiety, depression, and obsessive-compulsive behavior in health care workers and other workers may enhance our understanding of COVID-19 mental health needs. Research is needed to understand more fully the relationship between worker type, outbreak phase, and mental health changes over time, as well as the utility of validated tools in health care workers and other workers during pandemics. Our findings underscore the importance of anticipating and mitigating the mental health effects of pandemics using integrated implementation strategies. Finally, we demonstrate the ease of safely and rapidly assessing mental health needs using an SMS text messaging platform during a pandemic.

**International Registered Report Identifier (IRRID):**

RR2-10.2196/19292

## Introduction

Significant public health efforts have been implemented to contain the spread of SARS-CoV-2 [1] and mitigate its far-reaching impacts [2], including the negative psychological effects of the COVID-19 pandemic. After its first recognition in December 2019, COVID-19 spread rapidly, leading to it being declared a Public Health Emergency of International Concern, and later, a global pandemic, by the World Health Organization (WHO) [3]. Confirmed COVID-19 cases reached 1 million on April 1, 2020, doubling again only 9 days later [4]. Despite widespread efforts to “flatten the curve,” predictions on the ultimate resolution of the pandemic remain elusive due to variance in geographic disease burden, physical distancing requirements, and outbreak phase, among other factors [5], both within and beyond Canadian borders.

As knowledge of the COVID-19 pandemic increases and multilevel emergency response plans are activated [3], the implementation of containment and mitigation measures to stop the spread of COVID-19 continues to vary greatly by outbreak phase and between jurisdictions [5]. Unfortunately, pandemic containment and mitigation measures [6-8], while effective in limiting COVID-19 disease transmission, also likely contribute to negative psychological sequelae of affected individuals. The dynamic and rapidly changing circumstances related to the pandemic are creating uncertainty, stress, and anxiety, among other known adverse psychosocial correlates of the pandemic [9-14].

The negative impact of COVID-19 on global mental health is clear and increasing [9,13-26]. From a societal perspective, the emergent mental health burdens in health care workers [11-13,16,18,24-31] and other workers is of significant concern to the sustainment of health care services, as well as the maintenance of critical goods, services, and supply chains in other economic sectors.

While there is a dearth of research comparing health care workers’ mental health symptoms to that of other workers, especially during outbreaks, emerging research into health care worker mental health during the COVID-19 pandemic has documented high rates of depression [11,18,24,27-29], anxiety [11-13,18,24,26-29], stress [27-29], and other mental health conditions, including fear, insomnia [24], grief [25], posttraumatic stress disorder somatization [25], obsessive-compulsive symptoms [11,13,28], and vicarious traumatization [20]. Similar effects for health care workers were found in reviews and studies assessing the effects of past outbreaks [32-41].

Multiple studies of mental health symptoms in the general public during the current pandemic and past outbreaks also show evidence of depression [9,11,14,15,23,24,33,34,37,42,43], anxiety [11,15,23,24,33,42-44], stress [15,23,43], and other mental health conditions [15], including posttraumatic stress disorder [37,42,44-46], distress [14,33,37], fear [33,44,46], guilt, anger, and vicarious traumatization [20], conditions that can exacerbate panic or hysteria reactions [47]. Despite similar trends in both health care workers and the general population, the extent to which the mental health symptoms of health care workers and other workers differ is unclear. Notably, similar effects are also reported by individuals after exposure to other natural disasters and negative social conflict events including emergencies, terrorist attacks, earthquakes, and other calamities [9,10,17,32-37,42,48,49].

Negative effects on mental well-being also occur more frequently in those with pre-existing mental health concerns [23,26,39,41], which has important implications during the COVID-19 pandemic [47,50]. In Canada, approximately 75.0% of those who accessed health services for a mental illness sought help for mood and anxiety problems. In Alberta in particular, a relatively high proportion of Albertans are known to experience mood and anxiety disorders (eg, 12.9% for females and 6.4% for males) [51].

Studies that describe, assess, and compare the mental health symptoms of health care workers and other workers during the COVID-19 pandemic can offer service planners, policy makers, and leaders important insights about how best to mitigate mental health risks and preserve worker health and well-being. Ultimately, such research can help protect the sustained delivery of health care services, goods, and services in other key economic sectors, as well as maintain supply chains.

Unfortunately, adequate resources to limit negative psychological effects are almost invariably lacking during pandemics and other crises [18,39,40,52,53], evidenced by the mental health and well-being lessons emerging early in the COVID-19 pandemic. Attention to mental health and well-being is commonly superseded by urgent physical and public health needs during an emergency (ie, immediately caring for people with the disease, as well as monitoring, testing, and preventing further disease burden). However, systematic integration of mental health and well-being supports into the overall pandemic response may effectively mitigate and/or prevent health care and other worker, patient, and public harms, avoid burnout, and further help preserve the long-term stability and effectiveness of health care systems [31,45,50,54,55]. Although mental health harms are often noted or alluded to in pandemic guidance, there is a real need and opportunity to define practical measures guiding health care professionals in the psychological care of patients with COVID-19 and go beyond mere calls to action [50,56]. The sustainable delivery of goods and services in health care and other sectors is contingent on worker health and well-being. It is essential to understand if and how worker mental health varies during pandemics so that adequate supports are put in place.

Given the known risks associated with declining mental health correlates from past emergency situations and emerging COVID-19 studies, it is critical to understand the needs of health care workers and other essential workers and offer them appropriate, digital, and timely access to mental health supports. Within this context, we launched Text4Hope, a cognitive-behavioral therapy (CBT)–based SMS text messaging service that delivers once-daily supportive messages to support individuals’ mental health during the COVID-19 pandemic. The program offers support-seeking subscribers help identifying and modifying pandemic-related negative thoughts, feelings, and behaviors through texted advice and encouragement regarding the development of coping and resiliency skills [57]. The program was modeled after the Text4Mood program, a pre-existing, effective, evidence-based, and low-cost SMS text messaging intervention, developed as a therapeutic adjunct to support and optimize the mental health and treatment outcomes of people with mental health and addictions concerns residing in Alberta, Canada [58-62].

This study is an effort to better understand the mental health symptoms of health care workers and other workers during the COVID-19 pandemic, and to contribute evidence for mental health service planning. The specific aim of the study was to explore self-reported perceived stress, anxiety, depression, and obsessive-compulsive symptoms in health care workers and other workers who sought mental health support by subscribing to Text4Hope during the early phase of the COVID-19 pandemic.

## Methods

A cross-sectional survey was used to explore the self-reported prevalence of perceived stress, anxiety, depression, and obsessive-compulsive symptoms in health care workers and other workers who subscribed to Text4Hope.

### Recruitment

Text4Hope was offered to Alberta residents in the early stages of the COVID-19 pandemic through government and provincial health care delivery service news releases, email notifications, and website [57] postings related to COVID-19. Albertans seeking mental health support were invited to join the 3-month program by texting “COVID19HOPE” to a short code number. Subscription triggered a welcome text message containing a 5-10 minute online survey link requesting demographic characteristics (gender, age, ethnicity, education, relationship status, employment, type of employment [health care or other], housing status, and isolation status) and clinical characteristics (self-reported perceived symptoms of stress, anxiety, depression, and contamination- and cleanliness-associated obsessive-compulsive behaviors).

Clinical characteristics were assessed using validated screening scales for self-reported symptoms, including the Perceived Stress Scale (PSS; PSS score ≥14 indicates moderate or high stress) [63], the Generalized Anxiety Disorder 7-item (GAD-7) Scale (GAD-7 score ≥10 indicates likely generalized anxiety disorder [GAD]) [64], the Patient Health Questionnaire-9 (PHQ-9; a score ≥10 indicates likely major depressive disorder [MDD]) [65], and two items on the Brief Obsessive-Compulsive Scale (BOCS; pertaining to worry about dirt, germs, and viruses and handwashing behavior) [66]. Validated scales were chosen to better understand support seekers’ self-reported symptoms, potential symptom severity, and to screen for symptomatology. These scales were not intended as diagnostic tools.

Participant consent was indicated via submission of subscribers’ survey responses. The continuation of participation in the Text4Hope program was not dependent on the completion of the survey by participants; this was clearly stated to subscribers in the SMS text messaging program information provided at subscription. Ethical approval for the research was obtained through the University of Alberta Health Research Ethics Board (Pro00086163).

Based on a provincial population estimate of approximately 4.3 million people, the necessary sample size to generate prevalence estimates was 4157, assuming a 99% confidence level and 2% error. Previous research employing similar methodology in Alberta generated a 20% response rate [61]. Therefore, we planned to extract and analyze data after obtaining a minimum recruited sample of 20,785 Text4Hope subscribers.

### Analysis

Data analysis was undertaken using SPSS Statistics for Windows (Version 26; IBM Corp) [67]. Demographic characteristics of employed respondents were summarized by absolute numbers and percentages, by employment category (health care workers and other workers). Chi-square analysis with two-tailed significance (*P*<.05) was performed to assess the statistical differences between health care workers and other workers by their clinical characteristics. Variables with more than three responses in the chi-square test were subjected to post hoc analysis using adjusted residuals, with *z* scores and *P* values reported.

To assess the impact of “worker type” on self-reported symptoms of moderate or high stress, likely GAD, and likely MDD while controlling for demographic characteristics and isolation status, we entered all demographic predictors along with “worker type” into a logistic regression model. Correlation analysis was performed before the logistic regression analysis to rule out very strong correlations among predictor variables. Odds ratios from the binary logistic regression analysis were examined to determine the association between “worker type” and the likelihood of respondents self-reporting symptoms of moderate or high stress, likely GAD, and likely MDD, controlling for the other variables in the model. There were no imputations for missing data and the results presented reflect completed responses from the survey.

## Results

### Overview

During the first 6 weeks after launching the program (March 23 to May 4, 2020), a total of 44,992 subscribers joined Text4Hope; this data was extracted for analysis and is presented here. Of the 44,992 subscribers, 8267 responded to the online survey invitation, yielding a 19.4% response rate. Overall, 5990 (72.5%) respondents reported current employment, 958 (11.6%) reported current unemployment, 454 (5.5%) were students, 559 (6.8%) were retired, and 72 (0.9%) did not indicate their employment status. Of those who indicated they were employed, 1414 (23.6%) indicated health care industry employment and 3951 (66.0%) indicated employment in other industries. The remaining 625 (10.4%) of employed respondents did not indicate an industry affiliation.

The 6-week prevalence rates in the sample (n=8267) for moderate or high stress, likely GAD, and likely MDD were 85.6%, 47.0%, and 44.0%, respectively. Descriptive demographic characteristics of survey respondents, by worker type, are displayed in Table 1. There was no imputation for missing data and the data analyzed and displayed were from the data set remaining after cases with missing data were excluded.

Of employed support-seeking respondents, over 90.0% were aged ≥26 years (n=4939). Of this group, more than half were aged ≥41 years (n=2888) (Table 1). Most respondent workers identified as female (n=4621, 86.2%), were Caucasian (n=4441, 83.1%), were married, cohabiting, or partnered (n=3974, 74.2%), reported completion of postsecondary education (n=4814, 89.9%), and owned their own home (n=3836, 72.2%). There were statistically significant differences between health care workers and other workers on all demographic variables and isolation status. Health care workers had higher proportions of self-reported postsecondary education and home ownership, and higher rates of married, cohabiting, or partnered relationship status than other workers.

**Table 1:** Demographic characteristics of employed survey respondents.

Demographics and characteristics		Health care workers (N=1414)	Other workers (N=3951)	*P* value	Chi-square test result	Degrees of freedom	Total, n (%)
**Sex**				<.001	53.10	2	N/A^a^
	Male	105 (7.4)	588 (14.9)	N/A	N/A	N/A	693 (12.9)
	Female	1298 (91.1)	3323 (84.2)	N/A	N/A	N/A	4621 (86.2)
	Gender diverse	9 (0.6)	37 (0.9)	N/A	N/A	N/A	46 (0.9)
**Age (years)**				.003	13.80	3	N/A
	≤25	60 (3.4)	272 (7.0)	N/A	N/A	N/A	332 (6.3)
	26-40	540 (38.8)	1511 (38.9)	N/A	N/A	N/A	2051 (38.9)
	41-60	714 (51.4)	1915 (45.3)	N/A	N/A	N/A	2629 (49.9)
	>60	76 (5.5)	183 (4.7)	N/A	N/A	N/A	259 (4.9)
**Ethnicity**				.01	11.05	3	N/A
	Caucasian	1175 (83.3)	3266 (83.2)	N/A	N/A	N/A	4441 (83.1)
	Indigenous	37 (2.6)	140 (3.6)	N/A	N/A	N/A	177 (3.3)
	Asian	94 (6.7)	188 (4.8)	N/A	N/A	N/A	282 (5.3)
	Other	107 (7.4)	333 (8.5)	N/A	N/A	N/A	438 (8.2)
**Education**				<.001	90.71	3	N/A
	Less than high school diploma	6 (0.4)	81 (2.1)	N/A	N/A	N/A	87 (1.6)
	High school diploma	38 (2.7)	382 (9.7)	N/A	N/A	N/A	240 (7.8)
	Postsecondary	1358 (96.3)	3456 (87.6)	N/A	N/A	N/A	4814 (89.9)
	Other education	8 (0.6)	28 (0.7)	N/A	N/A	N/A	36 (0.7)
**Relationship status**				<.001	21.73	4	N/A
	Married, cohabiting, or partnered	1098 (77.2)	2876 (72.9)	N/A	N/A	N/A	3974 (74.2)
	Separated or divorced	116 (8.2)	296 (7.5)	N/A	N/A	N/A	412 (7.7)
	Widowed	9 (0.6)	29 (0.7)	N/A	N/A	N/A	38 (0.7)
	Single	183 (13.0)	715 (18.1)	N/A	N/A	N/A	898 (16.8)
	Other	7 (0.5)	30 (0.8)	N/A	N/A	N/A	37 (0.7)
**Housing status**				<.001	28.74	3	N/A
	Own home	1083 (77.2)	2752 (70.4)	N/A	N/A	N/A	3836 (72.2)
	Living with family	51 (3.6)	242 (6.2)	N/A	N/A	N/A	294 (5.5)
	Renting	259 (18.4)	892 (22.8)	N/A	N/A	N/A	1115 (21.7)
	Other	10 (0.7)	21 (0.5)	N/A	N/A	N/A	31 (0.6)
Self-isolated or self-quarantined		303 (21.7)	681 (17.5)	<.001	12.20	1	984 (18.6)

^a^N/A: not applicable.

### Univariate Analysis

In Table 2, we assessed the association between worker type and perceived stress, likely GAD, and likely MDD. Table 2 suggests that other workers reported higher moderate or high stress, higher likely GAD, and higher likely MDD compared to health care workers, with small effect sizes for each condition.

**Table 2:** Chi-square test of association between worker type, perceived stress, likely generalized anxiety disorder, and likely major depressive disorder.

Psychological symptom	Health care worker, n (%)	Other worker, n (%)	*P* value	Effect size (φ)
Perceived stress (moderate or high stress^a^)	1079 (81.7)	3149 (85.6)	<.001	0.05
Likely generalized anxiety disorder^b^	461 (38.1)	1600 (47.7)	<.001	0.09
Likely major depressive disorder^c^	401 (32.1)	1490 (43.6)	<.001	0.10

^a^Moderate or high stress was defined as a Perceived Stress Scale score ≥14.

^b^Likely generalized anxiety disorder was defined as a Generalized Anxiety Disorder Scale score ≥10.

^c^Likely major depressive disorder was defined as a Patient Health Questionnaire score ≥10.

### Logistic Regression

To assess the impact of “worker type” on moderate or high stress, likely GAD, and likely MDD while controlling for demographic characteristics and isolation status, we entered all seven characteristics in Table 1 and “worker type” into a logistic regression model.

For moderate or high stress, the full model containing all eight predictors was significant (*Χ*^2^[df=20, N=4874]=235.25, *P*<.001), suggesting the model was able to distinguish between respondents who reported moderate or high stress and those who did not. The model explained between 4.7% (Cox and Snell *R*^2^) and 8.2% (Nagelkerke *R*^2^) of the variance and correctly classified 84.7% of all cases. Controlling for all demographic characteristics and isolation status, health care worker type made a unique statistical contribution (Wald=8.44, *P*<.01) to the likelihood that a respondent presented with moderate or high stress. Other workers were 1.3 times more likely to report moderate or high stress during the COVID-19 pandemic compared to health care workers when all demographic variables and isolation status were controlled for (OR 1.30, 95% CI 1.10-1.55).

For likely GAD, the full model containing all eight predictor variables was significant (*Χ^2^*[df=20, N=4454]=364.75, *P*<.001), meaning the model was able to distinguish between respondents who had likely GAD and those who likely did not have GAD. The model explained between 7.9% (Cox and Snell *R*^2^) and 10.5% (Nagelkerke *R*^2^) of the variance and correctly classified 62.5% of all cases. Controlling for all demographic characteristics and isolation status, health care worker type made a unique statistical contribution (Wald=21.62, *P*<.001) to the likelihood of respondents meeting the cutoff threshold for likely GAD. Other workers were 1.4 times more likely to meet the cutoff threshold for likely GAD during the COVID-19 pandemic compared to health care workers when all demographic variables and isolation status were controlled for (OR 1.40, 95% CI 1.22-1.62).

For likely MDD, the full model containing all eight predictors was significant (*Χ*^2^[df=20, N=4 535]=341.33, *P*<.001), implying the model was able to distinguish between respondents who had likely MDD and those who likely did not have MDD. The model explained between 7.3% (Cox and Snell *R*^2^) and 9.8% (Nagelkerke *R*^2^) of the variance and correctly classified 63.9% of all cases. Controlling for all demographic characteristics and isolation status, health care worker type made a unique statistical contribution (Wald=27.79, *P*<.001) to the likelihood that respondents presented with likely MDD. Other workers were 1.5 times more likely to meet the cutoff threshold for likely MDD during the COVID-19 pandemic compared to health care workers when all demographic variables and isolation status were controlled for (OR 1.47, 95% CI 1.30-1.71).

As shown in Table 3, there were statistically significant associations between worker type and the tendency to worry about dirt, germs, and viruses and the tendency to wash hands repeatedly or in a special way due to fears of contamination. Post hoc analysis using adjusted residuals demonstrated that health care workers were significantly less likely to worry about dirt, germs, and viruses since the start of the COVID-19 pandemic compared to other workers (53.9% versus 62.5%, respectively; *z*=5.3, *P*<.001). Conversely, the proportion of health care workers who were worried about dirt, germs, and viruses before the COVID-19 pandemic was significantly higher than other workers (28.2% versus 22.1%, respectively; *z*=4.3, *P*<.001). Though the proportion of health care workers reporting compulsive hand washing due to fears of contamination after the COVID-19 pandemic began was lower than the proportion of other workers reporting the same (46.1% versus 57.6%, respectively; *z*=7.0, *P*<.001), a significantly higher proportion of health care workers engaged in compulsive hand washing before the pandemic due to fears of contamination (35.3% versus 29.3%, respectively; *z*=3.9, *P*<.001). In sum, the self-reported prevalence of obsessive-compulsive symptoms (ie, pre-existing worry and compulsive handwashing behavior) was higher in health care workers than in other workers, while the self-reported prevalence of worry about hand contamination and compulsive hand washing after the COVID-19 pandemic began was higher in other workers compared to health care workers.

**Table 3:** Chi-square test of association between worker type and manifestation of obsessive-compulsive symptoms.

Clinical variable		Health care workers, n (%)	Other workers, n (%)	*P* value	Effect size (φ)
**Worried about dirt, germs, and viruses**				<.001	0.08
	Only since the COVID-19 pandemic	663 (53.9)	2137 (62.5)	N/A^a^	N/A
	Before and during the COVID-19 pandemic	347 (28.2)	756 (22.1)	N/A	N/A
	Never	220 (17.9)	525 (15.4)	N/A	N/A
**Wash hands repeatedly or in a special way due to fears of contamination with dirt, germs, and viruses**				<.001	0.11
	Only since the COVID-19 pandemic	566 (46.1)	1969 (57.6)	N/A	N/A
	Before and during the COVID-19 pandemic	433 (35.3)	1002 (29.3)	N/A	N/A
	Never	229 (18.6)	447 (13.1)	N/A	N/A

^a^N/A: not applicable.

## Discussion

### Overview

This is the first large-sample, cross-sectional Canadian study to examine the self-reported prevalence of stress, anxiety, depression, and obsessive-compulsive symptoms by worker type during the COVID-19 pandemic. We used an online survey to gather self-reported symptoms of stress, anxiety, depression, and obsessive-compulsive behaviors in support-seeking individuals subscribing to the Text4Hope daily SMS text messaging support program. Text4Hope sent once-daily supportive text messages to provide Alberta residents with advice and encouragement to develop coping skills and optimize resiliency during the COVID-19 pandemic.

### Principal Results

Our study sample comprised support-seeking individuals who subscribed to Text4Hope and had self-reported elevated symptomatology. The proportion of employed respondent subscribers (n=3721, 72.2%) was comparable to the 2017 Alberta employment rate (66.7%), the full-time employment rate for females (73.3%), and the proportion of female Albertans employed in the health care/social assistance sector, particularly in the ≥45 years age cohort (79.5%) [68]. Respondent demographics were comparable to only some Alberta resident worker characteristics (ethnicity, housing status) and Alberta health care worker characteristics (age, ethnicity, gender, education, and housing status) [69,70].

Health care worker demographic characteristics were comparable to surveyed health care worker characteristics in other international jurisdictions during the COVID-19 pandemic [11,12,18,24].

We found that the overall self-reported 6-week prevalence of moderate or high stress, likely GAD, and likely MDD symptoms were higher than baseline [51] during the early COVID-19 outbreak phase and were statistically significantly higher in other workers than in health care workers.

The prevalence of pre–COVID-19 worry and compulsive handwashing behavior was higher in health care workers than in other workers, while worry and compulsive handwashing behavior after the COVID-19 pandemic began was significantly higher in other workers than in health care workers.

### Limitations

Our study has several important limitations. The prevalence of self-reported stress, anxiety, depression, and obsessive-compulsive symptoms was assessed in health care workers and other workers who voluntarily sought support by subscribing to Text4Hope. Despite demographic similarities, voluntary subscribers seeking support may differ significantly from the nonsubscribing employed or general employed population of Alberta, thereby influencing self-reported symptom prevalence rates. In addition, an overall elevated symptomatology, while higher than that of the general population, would be expected given that survey respondents were a sample of mental health support-seeking subscribers. Not all respondents answered every survey question, resulting in missing data for multiple variables and reduced sample sizes, thereby potentially reducing the generalizability of the findings.

Given both the dearth of comparative evidence on mental health burden in workers and the limitations of our study design, we were unable to explore subtle dynamics related to subtypes of health care workers and other workers (eg, frontline versus nonfrontline, high-risk occupations in the “other” category). Similarly, we were unable to further refine our understanding of other pandemic dynamics that likely occurred during the study period, such as fluctuating employment status, disease exposure risk, and worker access to and use of personal protective equipment. This study is limited by its inability to address these factors, which nonetheless ought to be considered in comparisons with other, related literature.

There are several limitations related to the use of screening tools. Logistics, sample size, cost, resources, and time factors precluded the use of diagnostic interviews; our use of screening scales estimated self-reported symptom burden and was not intended to yield mental health diagnoses. However, a screening tool is appropriate in the context of capturing data quickly at a general population level and was thus the tool indicated for use in the current study.

In addition, it should be noted that it is unclear the extent to which the BOCS screening tool is valid for, and can differentiate the range of adaptive-to-obsessive-compulsive behaviors during a pandemic. For example, consistent and repeated handwashing has been emphasized as an important means of avoiding COVID-19 transmission.

Finally, we assessed self-reported symptoms during an early pandemic phase. It is possible that the timing of these measurements (and/or other contextual features that we did not study) were maligned with studies measuring the same outcomes during different pandemic phases and could bias the interpretation of our findings.

### Comparison With Prior Work

Several studies of health care worker mental health during the COVID-19 pandemic revealed higher stress [11-13,18,26,29], anxiety [18,24,29], depressive [11,12,18,24], and obsessive-compulsive [11] symptoms; these results align well with our 6-week prevalence findings. These studies also found that heightened stress, anxiety, and depressive symptoms were consistently more pronounced in females, and in those with increased exposure to afflicted individuals (eg, frontline health care workers, close proximity to higher intensity outbreak conditions). Although our 6-week prevalence findings matched this overall increase in health care worker mental health burden during an outbreak, higher levels of stress, anxiety, and depressive symptoms were also reported by other workers in our study.

Based on existing evidence, there may be several plausible explanations for our findings, although their interpretation should be approached with caution, given the lack of published studies directly comparing mental health symptoms by worker type, particularly during COVID-19.

There are many possible factors contributing to mental health concerns among the general public during times of societal unrest, as documented during previous infectious disease outbreaks [32,38-42], emergencies, disasters [15-17,19,44,62], and the current pandemic [18,38,71]. Within the Alberta context, several of these factors (eg, high unemployment [69], job uncertainty, strict confinement, and mitigation measures, as well as pre-existing, unaddressed mental health concerns arising in part from economic downturn and natural disasters [51,62,72]) or other contextual factors may be contributing to the increased mental health symptoms in other workers. These factors may be more prevalent in other workers who actively sought support by subscribing to Text4Hope, contributing to a higher symptom burden in this worker group. Current trends in Alberta align with this hypothesis: a recent Mental Health Index survey of 3000 Canadians [73] showed Alberta residents experienced the highest national month-over-month decline in Mental Health Index scores. Decreases were observed in all Index subscores (including risk measures for anxiety, depression, work productivity, optimism, and isolation, in decreasing order of magnitude), with greater declines among the unemployed, females, and younger individuals. Albertans had the second highest reported Mental Stress Change Score across Canada [73]. Nonetheless, our observation of elevated symptomatology overall might be anticipated given that respondents were a sample of mental health support-seeking Text4Hope subscribers.

Alternatively, it is also possible that the health care workers seeking support in our study sample were protected from mental health harms due to the influence of other documented but unassessed modifiers such as the following: ready access to relevant knowledge, training, protocols, timely information, personal protective equipment, and a support network of peers experiencing similar stress, as well as coordinated efforts, clear communication, and other occupational and/or social supports [12,13,41,53,55,74]. Past research shows the presence of these and other precautionary measures may help health care workers feel a heightened sense of certainty or control over their situation. Perhaps support-seeking health care worker respondents’ pre-existing knowledge and training served to reinforce their roles and practice, provided comfort and enhanced resiliency, and helped mitigate the negative effects of the pandemic as well as subsequent confinement and mitigation strategies [12,13,35,38,39,41,45,55].

Such a resilience hypothesis in health care workers would contrast with recent COVID-19–related studies by Lai and colleagues [18] and Huang and colleagues [24], which demonstrated an increased risk of negative mental health sequelae in females [11-14,18,27,29], nurses [11,12,14,18,27,29], frontline staff, and those with closer proximity to higher intensity outbreak working conditions [11,12,14,18,23,25,26,55]. Similar findings from past outbreaks also implicated concerns about health of self and family, disease spread, adequate supplies/resources, an influx of suspected cases, occupational changes, pre-existing chronic disease, isolation, and feelings about isolation and vulnerability as key negative contributing factors [18,38,41,75]. Zhang and colleagues’ recent survey comparing medical and nonmedical health care worker mental health symptoms in China post–COVID-19 showed higher prevalence and total scores for insomnia, anxiety (GAD-2), depression (PHQ-2), somatization, and obsessive-compulsive (Symptom Checklist 90-Revised) symptoms in health care workers [11]. Similar findings for anxiety occurred in a mixed sample survey undertaken during the COVID-19 pandemic [13]. Additionally, a quasi-comparator study of mental health concerns in frontline workers and administrative health care workers found frontline workers were 1.4 times more likely to feel fear and 2 times more likely to suffer anxiety and depression than their administrative (ie, nonfrontline, low-risk exposure) counterparts (despite lower relative exposure, both worker groups were health care sector employed, making clear comparisons difficult) [11,13,28]. Lastly, the most relevant large post–COVID-19 pandemic web-based survey by Huang and Zhao [24] failed to find any statistically significant differences between health care and other workers’ prevalence of anxiety or depressive symptoms, and only a higher prevalence of sleep disruptions in health care workers.

Clearly, large longitudinal studies that specifically compare the symptoms, risk exposure [18], and other contextual correlates (eg, pre-existing knowledge of disease and disease transmission, outbreak phase, and geographic proximity to high risk situations) of health care workers and other workers are needed. Ongoing evidence syntheses would also help to clarify and explain these apparent discrepancies in the future.

Our self-reported 6-week prevalence rates for likely GAD (47.0%) and likely MDD (44.0%) were slightly higher and lower than symptom estimates reported by Lai et al [18] (44.6% GAD, 50.4% MDD), even considering the authors’ use of a slightly lower GAD-7 cutoff score (7). Our prevalence estimates for likely MDD (44.0%) were also higher than the estimates of moderate or severe depression symptoms reported by Kang et al (28.6%) [27] and Huang and Zhao (20.1%) [24] in the postpandemic period. Although these findings may reflect true mental health burden differences, it is possible that the timing of symptom measurement during a pandemic influences symptom severity, particularly if the implementation of confinement and mitigation measures varies as widely as during the COVID-19 pandemic [5]. This study captured 6-week prevalence in an early pandemic phase, whereas Lai et al measured the same burden during the pandemic. Kang and colleagues and Huang and Zhao both measured health care worker symptoms in the postpandemic phase. It is also possible that jurisdictions most rapidly affected by the pandemic have less knowledge, less time to prepare and assess/address risk, and possess fewer supplies and resources, and thus may report higher mental health concerns [12,13]. Furthermore, despite the elevated psychopathology we observed, we note that respondents were a sample of mental health support-seeking Text4Hope subscribers and our findings should be considered in light of this context.

In our study, the prevalence of worry about dirt, germs, and viruses and compulsive handwashing behavior since the COVID-19 pandemic began was significantly higher in other workers than in health care workers. This finding is aligned with a population-based study by Wang et al [15] early in the COVID-19 pandemic, in which members of the general public reported newly adopting handwashing after touching contaminated objects (66.6%), washing their hands with soap (56.5%), and always washing their hands after coughing, sneezing, or rubbing their nose (41.0%). These authors found that handwashing behavior was linked to lower mental health symptom scores, suggesting they had a protective effect during the early stages of the pandemic [15]. This finding aligns with our observation of lower reported worry in other workers before and during the COVID-19 pandemic, and their subsequent adoption of compulsive handwashing behavior since the onset of the COVID-19 pandemic.

With respect to health care workers’ obsessive-compulsive symptoms, it is unclear the extent to which professional training; heightened attention to dirt, germs, and viruses; handwashing behavior, and disease transmission could appear maladaptive when assessed with the BOCS tool during a pandemic. Using a different validated scale, Zhang and colleagues [11] recently observed higher measures of obsessive-compulsive symptoms after the peak of the COVID-19 pandemic in frontline health care workers, which aligns with our finding of higher health care worker worry and handwashing due to fears of contamination before and during the pandemic. The BOCS tool was validated for use in adult psychiatric outpatients, not specifically for pandemics, nor for profession-specific groups, and therefore we advise caution in the interpretation of these findings. Certainly, the negative impacts of pervasive multimodal media and recurring widespread handwashing advice during COVID-19 are well documented [76] and particularly problematic for those with pre-existing obsessive-compulsive behaviors [77]. Further study is needed to understand how worry of contamination and compulsive handwashing behavior relate to worker type and timing of symptom assessment during outbreak phases. Future research is also needed to understand the extent to which worry and handwashing due to fears of contamination are adaptive during a pandemic, when assessed with validated scales.

### Conclusions

Demographic and clinical correlate data pertaining to mental health needs were successfully collected through Text4Hope and provided insight on the state of mental health concerns in health care workers and other workers.

Overall, our study findings mirrored much of the emerging literature documenting increased stress, anxiety, and depression symptoms arising during the current COVID-19 pandemic, and during previous infectious disease outbreaks. However, as compared to the literature and given our stated limitations, discrepant findings may be a function of measurement timing during a particular outbreak phase (early, interim, post), attributable to pre-existing context, and/or may be related to our study population, which was comprised of support-seeking individuals. A health care worker resilience hypothesis (eg, that health care workers are protected by knowledge, resources, supplies, messaging, and heightened certainty and control over workload [12,18]) is not well supported by our findings, nor the bulk of the existing and emergent COVID-19 pandemic literature pertaining to mental health concerns in this group. Furthermore, we note that the array of documented mental health risk factors, combined with higher proportions of females working in health care, community, caregiving, and domestic roles, seems to indicate an increased mental health vulnerability for females during COVID-19.

We observed support-seeking health care workers report significantly higher worry about contamination and compulsive handwashing symptoms prior to the onset of the COVID-19 pandemic and a higher proportion of other workers reporting these symptoms only since the onset of the pandemic. However, both of these findings should be interpreted with caution given it is unclear the extent to which validated scales accurately differentiate adaptive and maladaptive symptoms associated with worry about contamination and handwashing behavior in health care workers, or accurately differentiate symptoms during pandemic periods.

These findings add to our understanding of mental health needs during the COVID-19 pandemic; however, further research is required to understand and confirm in more detail the potential effects of a given outbreak phase on the measurement of mental health burden, the role of context, and the extent to which validated tools have utility in health care workers and in different worker groups during pandemics. The findings also underline the importance of anticipating and mitigating mental health needs as an integrated part of planning and confinement/mitigation strategy implementation [50,55,78-82] and highlight the ease with which current mental health needs can be safely assessed using a self-subscribing SMS text messaging platform. This study took place in a nation where the health care system is publicly funded, reasonably accessible to most residents, and operationally sound; however, it is possible that the mental health effects we observed could be further amplified, particularly for females, in nations where these circumstances are not the norm.

## Abbreviations

AHS: Alberta Health Services
BOCS: Brief Obsessive-Compulsive Scale
CBT: cognitive behavioral therapy
GAD: generalized anxiety disorder
GAD-7: Generalized Anxiety Disorder Scale, 7-item scale
MDD: major depressive disorder
PHQ-9: Patient Health Questionnaire, 9-item scale
PSS: Perceived Stress Scale
Text4Hope: Alberta Health Services Text for Hope program
WHO: World Health Organization
